# Correction: Research status, industrial application demand and prospects of phenolic resin

**DOI:** 10.1039/d0ra90066d

**Published:** 2020-06-12

**Authors:** Yanru Xu, Lifang Guo, Haonan Zhang, Huamin Zhai, Hao Ren

**Affiliations:** Jiangsu Provincial Key Lab of Pulp and Paper Science and Technology, Nanjing Forestry University Nanjing 210037 China renhao@njfu.edu.cn; Jiangsu Co-Innovation Center for Efficient Processing and Utilization of Forest Resources Nanjing 210037 China

## Abstract

Correction for ‘Research status, industrial application demand and prospects of phenolic resin’ by Yanru Xu *et al.*, *RSC Adv.*, 2019, **9**, 28924–28935. DOI: 10.1039/C9RA06487G.

The authors wish to draw the readers’ attention to their closely related paper written in Chinese, which was published at nearly the same time in *China Adhesives*,^1^ and should have been cited in this *RSC Advances* article. In their *RSC Advances* article, the authors included additional information about increasing the production and consumption data of phenolic resin in the global market. Further information about new technologies and modification research on phenolic resin (non-toxic resin, non-formaldehyde phenolic resin) was also included in the English article.

The Royal Society of Chemistry apologises for these errors and any consequent inconvenience to authors and readers.

## Supplementary Material
